# ATP-binding Cassette Transporters Substantially Reduce Estimates of ALDH-positive Cancer Cells based on Aldefluor and AldeRed588 Assays

**DOI:** 10.1038/s41598-019-42954-9

**Published:** 2019-04-23

**Authors:** Jin Won Park, Kyung-Ho Jung, Youngjoo Byun, Jin Hee Lee, Seung Hwan Moon, Young Seok Cho, Kyung-Han Lee

**Affiliations:** 1 Department of Nuclear Medicine, Samsung Medical Center, Seoul, Korea; 2 Department of Health Sciences and Technology, SAIHST, Sungkyunkwan University School of Medicine, Seoul, Korea; 3 College of Pharmacy, Korea University, Sejong, Korea

**Keywords:** Enzyme mechanisms, Cancer imaging, Cancer stem cells

## Abstract

Aldehyde dehydrogenase (ALDH) assays measure the accumulated fluorescence of enzyme products. However, cancer cells frequently co-express ALDH and ATP-binding cassette (ABC) transporters, which might mediate efflux of ALDH assay reagents. We demonstrate expression of active multidrug resistance protein1 (MDR1), multidrug resistance-associated protein (MRP), and breast cancer resistance protein (BCRP) in CT26 cancer cells as well as expression of MRP and BCRP in HT29 cancer cells. Without transporter inhibition, only small portions of both cell types were estimated to be ALDH-positive based on Aldefluor and AldeRed588 assays. However, MK-571 (MRP inhibitor) and novobiocin (BCRP inhibitor) substantially increased the rate of ALDH-positive CT26 cells based on either Aldefluor or AldeRed588 assays. Verapamil (MDR inhibitor) did not influence assay results. MK-571 also substantially increased the rate of ALDH-positive HT29 cells. Limiting dilution assays demonstrated greater numbers of tumor-spheres formed by Aldefluor-positive compared to -negative CT26 cells selected in the presence of MK-571 or novobiocin but not in their absence. These results reveal that Aldefluor and AldeRed588 products are efficient substrates for MRP- and BCRP-mediated efflux and substantially reduce estimated ALDH positivity rates in cancer cells. These findings demonstrate that complete blockade of these transporters is important to ensure accurate ALDH assay results and to develop newer assay techniques.

## Introduction

A major obstacle to curing cancer is treatment resistance. One mechanism for acquiring chemotherapeutic resistance is expression of ATP-binding cassette (ABC) transporters that mediate drug efflux^1,2^. Major ABC transporters expressed in cancer cells are multidrug resistance protein1 (MDR1; P-glycoprotein), breast cancer resistance protein (BCRP), and MDR-related protein (MRP). In stem cells, these transporters help maintain quiescence by stabilizing the internal milieu. ABC transporters are also highly expressed in cancer stem cells (CSCs), a highly tumorigenic population^3^, where they contribute to treatment resistance of cancers of the brain^4^, lung^5^, ovary^6^, prostate^7^, and nasopharynx^8^.

Given the crucial role of CSCs in tumor resistance and recurrence, targeting specific markers to recognize and isolate these cells has become an important tool for cancer research^9,10^. CSCs have several interesting characteristics, including the capacity for self-renewal and differentiation, high expression of organ-specific surface markers such as CD133, CD44, and CD29, and increased Notch and Wnt pathways^3^. High ALDH activity is widely used as a detection and isolation marker that is often expressed in CSCs from various organs^9,10^. This underlines the importance of identifying factors that influence the results of Aldefluor assays. ALDH belongs to a family of enzymes that catalyze NAD^+^ -dependent conversion of aldehydes to corresponding weak carboxylic acids^11^. ALDH1 is an established marker of cancer stemness that strongly correlates with self-renewal capacity^12^ and chemotherapy resistance in various cancer types^13,14^. Fluorescence-activated cell sorting (FACS) using fluorescent reagents is currently the standard method to identify cells that have increased ALDH activity. In these assays, ALDH mediates conversion of reagent substrates into products that can no longer diffuse out of cells. This leads to increased intracellular accumulation of fluorescent signals for detection.

Aldefluor kits are the most widely employed ALDH assay method. This technique uses Aldefluor (BODIPY-aminoacetaldehyde, BAAA), which is a fluorescent non-toxic ALDH substrate that freely diffuses in and out of intact cells. ALDH converts Aldefluor into negatively charged BODIPY-aminoacetate (BAA), which is retained inside cells and results in increased fluorescence.

However, many cancer cells with high ALDH activity co-express ABC transporters. This raises a potential problem for exploiting reagent accumulation to identify ALDH-positive cells. In fact, commercially available Aldefluor and AldeRed assay kit manuals state that verapamil, an MDR1 inhibitor, can be added to ALDH assays. A clear understanding of how Aldefluor and AldeRed588 reagents undergo efflux through ABC transporters is important to ensure accurate assays of cancer cell ALDH activity. Specific inhibitors that reverse the ability of ABC transporters to pump out cognate substrates can offer valuable information.

Specific MDR1, BCRP, and MRP inhibitors were used in this study to clarify the extent to which Aldefluor and AldeRed588 act as substrates for efflux out of cancer cells through ABC transporters. HT29 and CT26 colon cancer cells were used because colon cancer ALDH is associated with metastasis and poor prognosis^15^. While both cell lines have significant ALDH activity^16,17^, CT26 cells constitutively express the major ABC transporter, MDR1^18^, whereas HT29 cells express very little MDR1^19^. We further assessed how activities of these transporters reduce reagent retention and lead to underestimation of ALDH positivity based on assays using Aldefluor and AldeRed588.

## Results

### Major ABC transporter activity and expression in HT29 and CT26 cancer cells

**Figure 1:**
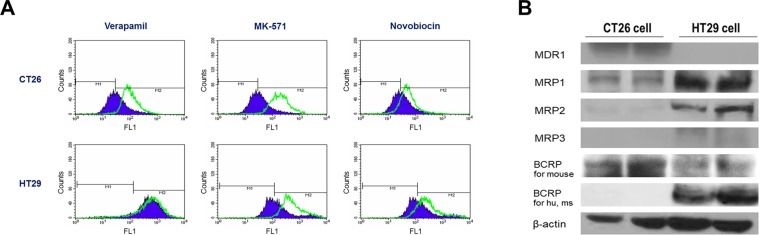
Efluxx-ID Green assay and Western blots for ABC transporter activity and expression level. (**A**) Verapamil, MK-571, and novobiocin were used as specific inhibitors of MDR1, MRP1/2, and BCRP activity, respectively. Histograms of untreated control CT26 and HT29 cells are shown in purple, while green lines indicate histograms obtained after treatment with verapamil, MK-571, or novobiocin. Right-shifting of the latter compared to the former histogram represents inhibition of efflux through the corresponding ABC transporter (representative of 3 samples per group). (**B**) Western blotting for MDR1, MRP1, MRP2, MRP3, and BCRP protein in CT26 and HT29 cells. For hu, ms = specific against human and mouse protein. Blots were cropped to combine into single figure. For full length blot pictures, see Supplementary Fig. S7.

In this study, verapamil, MK-571, and novobiocin were used as MDR1, MRP1/2, and BCRP inhibitors, respectively. Histograms of CT26 mouse colon cancer cells incubated with Efluxx-ID Green dye and analyzed by FACS showed a substantial right shift in the presence of verapamil and MK-571 and a significant but mild right shift in the presence of novobiocin (Fig. 1A). These results indicated that CT26 cells expressed MDR1, MRP, and BCRP that pumped out assay dye. Histograms of HT29 human colon cancer cells showed a substantial right shift due to MK-571 and a mild shift due to novobiocin, but no response to verapamil (Fig. 1A). This indicated expression of MRP and BCRP but not MDR1.

Since ABC transporters require energy in the form of ATP to translocate substrates across the cell membrane, transporter assays were also additionally compared on CT26 cells using PBS, RPMI media, and Hanks’ balanced salt solution (HBSS) containing 2% fetal bovine serum (FBS). The results were highly consistent using RPMI media or HBSS compared to phosphate buffered saline (PBS) (Supplementary Fig. S1), except for a slightly greater right shift with verapamil in RPMI media or HBSS compared to PBS.

ABC transporter expression was also evaluated at the protein level using Western blots, which confirmed MDR1 expression in CT26 cells but not in HT29 cells. Conversely, MRP1/2/3 were all highly expressed in HT29 cells but weakly expressed in CT26 cells. BCRP expression was also high in HT29 cells and CT26 cells (Fig. 1B).

### ALDH expression in HT29 and CT26 cancer cells

**Figure 2:**
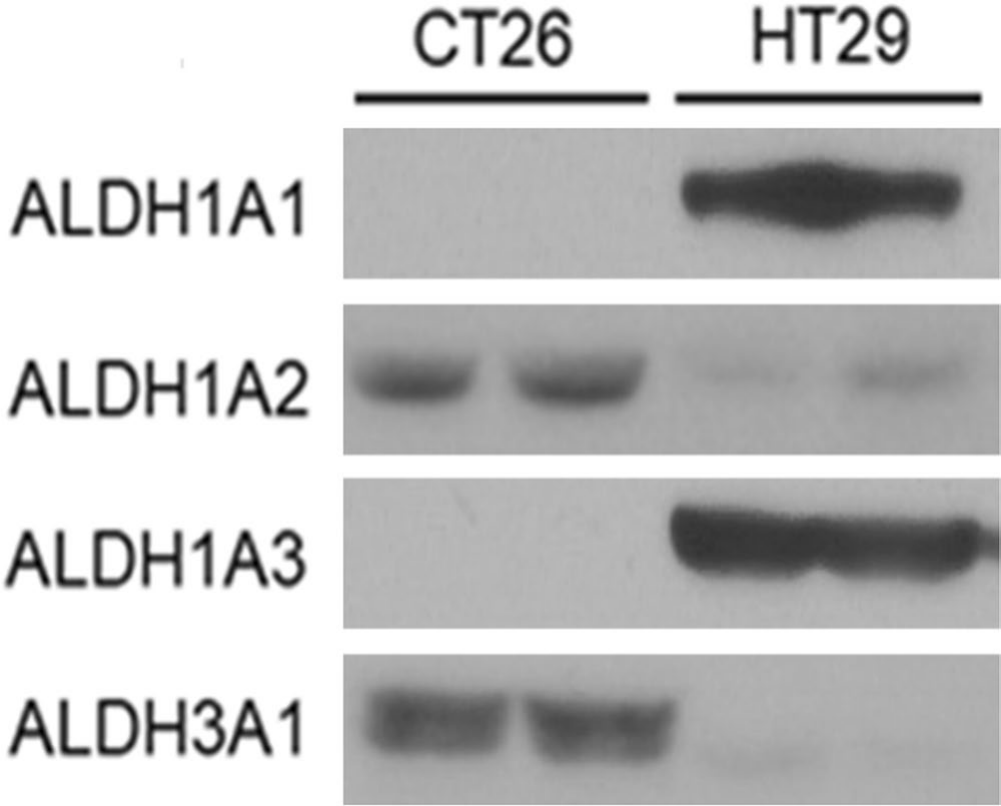
ALDH expression at protein level. Western blotting for ALDH1A1, ALDH1A2, ALDH1A3, and ALDH3A1 protein in CT26 and HT29 cells. Blots cropped for representative blots are shown. For full length blot pictures, see Supplementary Fig. S8.

Western blots showed that ALDH1A1 was highly expressed in HT29 cells but weakly expressed in CT26 cells. Among other subtypes that contribute to Aldefluor assays, ALDH1A2 was moderately expressed in both cell types; ALDH1A3 was highly expressed in HT29 cells, and ALDH3A1 was highly expressed in CT26 cells (Fig. 2).

### Influence of ABC transporter inhibitors on ALDH assays based on Aldefluor

Baseline FACS analysis of fluorescence signals from Aldefluor product accumulation was performed in PBS that did not contain any ABC transporter inhibitor. A specific ALDH inhibitor, diethylaminobenzaldehyde (DEAB), was used to correct for background fluorescence. The resultant proportion of Aldefluor-positive cells at baseline was 6.3 ± 2.4% for CT26 cells and 11.0 ± 5.2% for HT29 cells (Supplementary Fig. S2).

**Figure 3:**
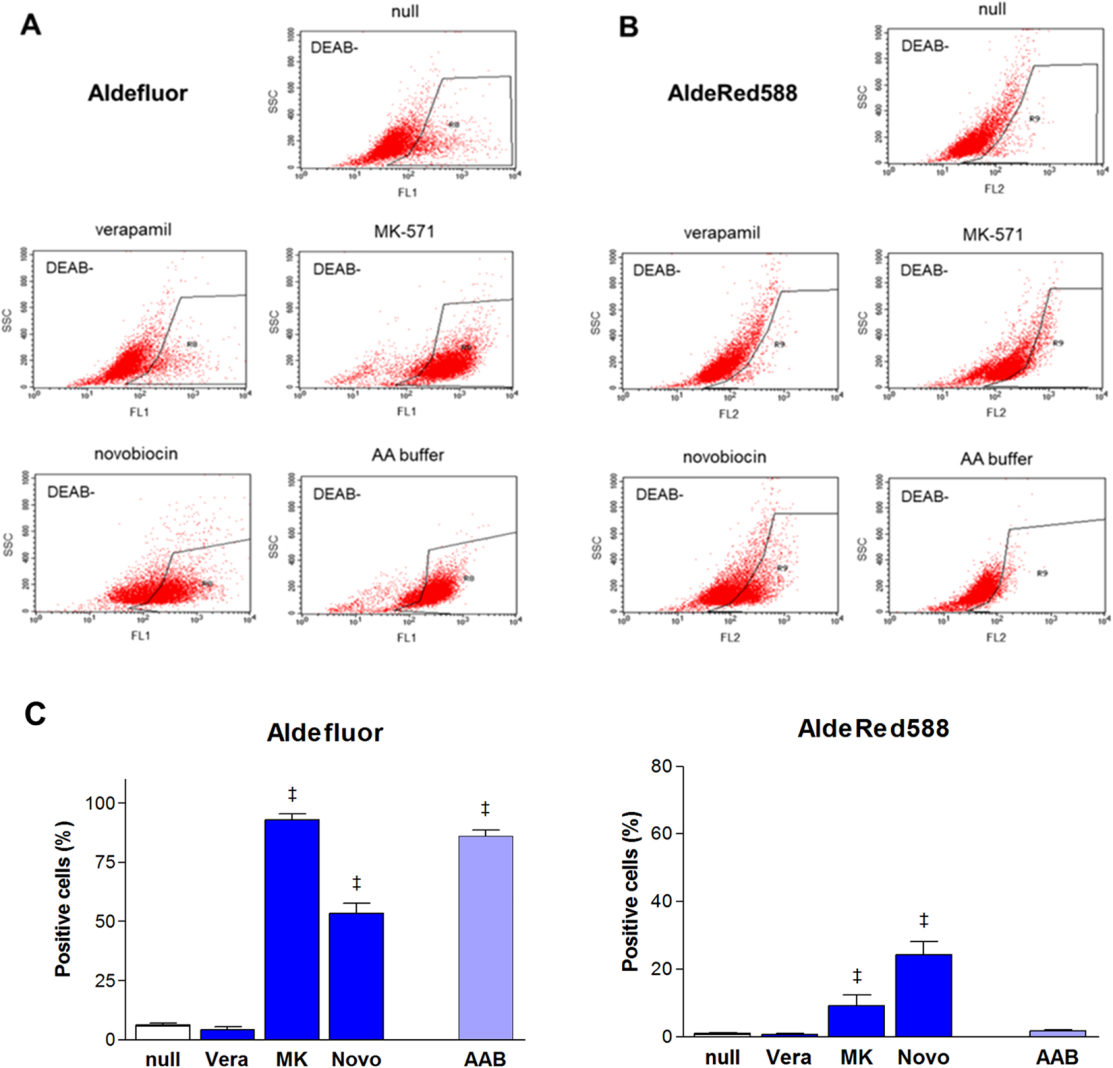
ABC transporter inhibitors affect Aldelfluor or AldeRed588 efflux from CT26 cells. FACS (representative of 4–6 samples per group) of Aldefluor (**A**) and AldeRed588. (**B**) ALDH assays of CT26 cells in the presence of MDR1 inhibitor (verapamil; Vera, 20 μM), MRP inhibitor (MK571; MK, 50 μM), and BCRP inhibitor (novobiocin; Novo, 200 μM) compared to their absence (null; top). DEAB was not used. ALDH assays of CT26 cells performed in Aldefluor assay buffer (AA buffer) compared to PBS with 2% FBS (null). ALDH-positive cells within black boxes are identified by low side scatter and high fluorescence. (**C**) Proportions of ALDH positive CT26 cells based on FAC analysis using Aldelfluor (top) and AldeRed588 fluorescence (bottom). Effects of ABC transporter inhibitors and use of Aldefluor assay buffer (AAB) are compared. Bars are mean ± SE of % relative to controls obtained from 2 or 3 independent experiments for 4–6 samples per group. ^‡^*P* < 0.001, compared to vehicle treated controls.

**Figure 4:**
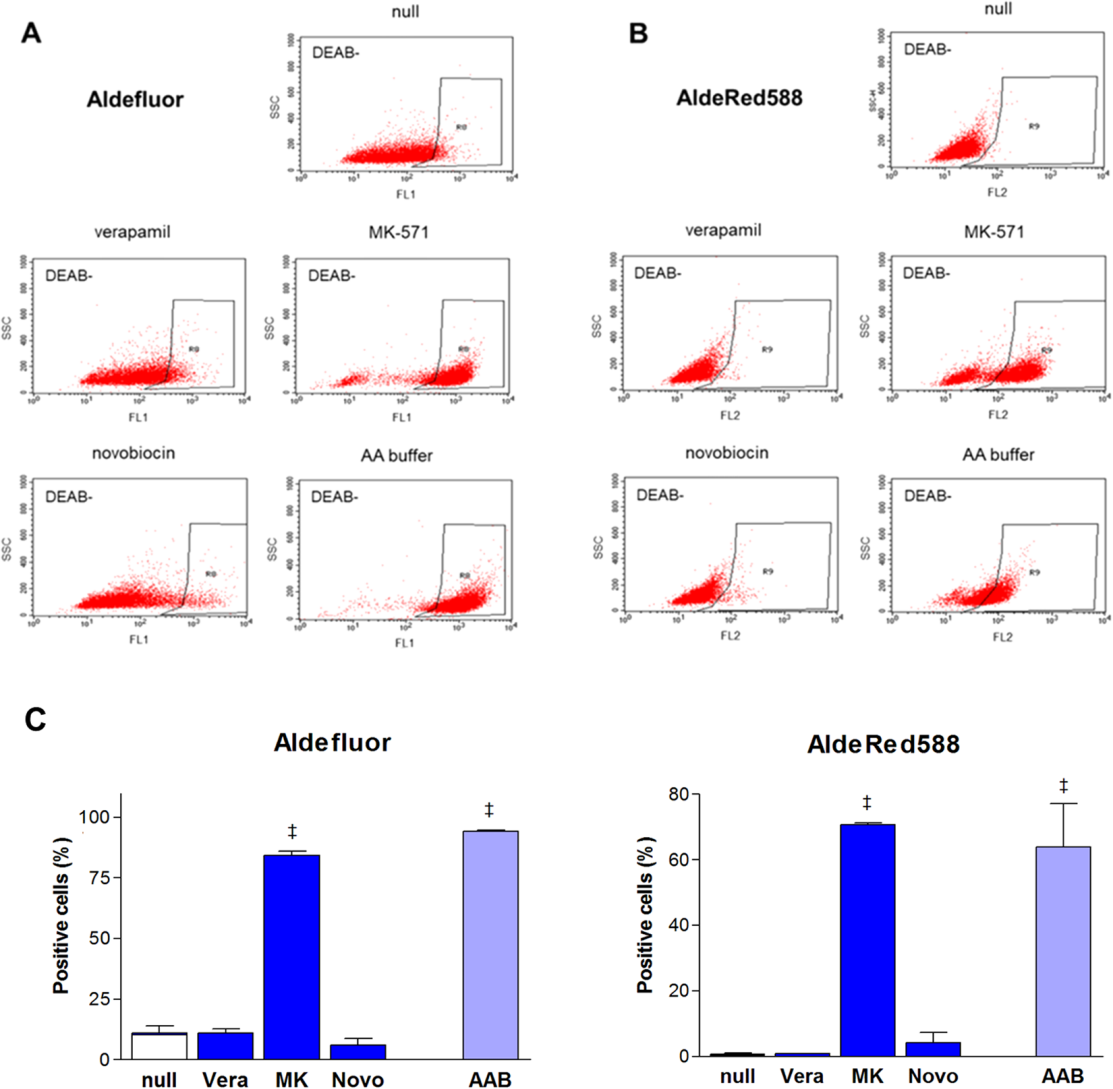
ABC transporter inhibitors affect Aldelfluor or AldeRed588 efflux from HT29 cells. FACS (representative of 4–6 samples per group) of Aldefluor (**A**) and AldeRed588. (**B**) ALDH assays of HT29 cells in the presence of MDR1 inhibitor (verapamil; Vera, 20 μM), MRP inhibitor (MK571; MK, 50 μM), and BCRP inhibitor (novobiocin; Novo, 200 μM) compared to their absence (null; top). DEAB was not used. ALDH assays of CT26 cells performed in Aldefluor assay buffer (AA buffer) compared to PBS with 2% FBS (null). ALDH-positive cells within black boxes are identified by low side scatter and high fluorescence. (**C**) Proportions of ALDH positive HT29 cells based on FACS analysis using Aldelfluor (top) and AldeRed588 fluorescence (bottom). Effects of ABC transporter inhibitors and use of Aldefluor assay buffer (AAB) are compared. Bars are mean ± SE of % relative to controls obtained from 2 or 3 independent experiments for 4–6 samples per group. ^‡^*P* < 0.001, compared to vehicle treated controls.

When FACS was repeated in the presence of verapamil, neither MDR1-positive CT26 cells (Fig. 3) nor MDR1-negative HT29 cells (Fig. 4) showed a change in accumulated fluorescence. This indicated that Aldefluor was not a substrate for MDR1-mediated reflux. In contrast, MK-571 substantially increased fluorescence in both CT26 (Fig. 3) and HT29 cells (Fig. 4). Thus, based on Aldefluor retention, MK-571 treatment markedly increased ALDH positivity from 6.3 ± 2.4% to 92.8 ± 4.4% for CT26 cells (*P* < 0.001) and from 11.0 ± 5.2% to 84.4 ± 2.4% for HT29 cells (*P* < 0.001). In Aldefluor assays performed with novobiocin, CT26 cells displayed a moderate increase in fluorescence (Fig. 3), whereas HT29 cells showed no effect (Fig. 4). Novobiocin increased ALDH positive CT26 cells from 6.3 ± 2.4% to 53.5 ± 5.8% (*P* < 0.001). Another specific BCRP inhibitor, Ko143, increased ALDH positivity to 35.7% for CT26 cells and 27.8% for HT29 cells (Supplementary Fig. S3).

Aldefluor assays performed in RPMI media and PBS were also compared with similar results (Supplementary Fig. S4). The only difference was fewer Aldefluor positive cells in the presence of novobiocin using RPMI compared to PBS. This raises the possibility that the concentration of novobiocin used was not sufficient to completely block BCRP activity in the presence of energy substrate-containing media.

### Influence of ABC transporter inhibitors on ALDH assays based on AldeRed588

Baseline FACS analysis using AldeRed588 resulted in low ALDH positive rates of only 1.1 ± 0.4% for CT26 cells and 0.7 ± 0.7% for HT29 cells (Supplementary Fig. S2). Addition of verapamil did not influence fluorescence accumulation in either cell type, indicating that AldeRed588 was also a poor substrate for MDR1-mediated reflux. MK-571 increased AldeRed588 retention in both cell types (Figs 3 and 4), resulting in a mild increase of ALDH-positive CT26 cells from 1.1 ± 0.4% to 5.9 ± 0.7% (*P* < 0.001) and a marked increase of ALDH-positive HT29 cells from 0.7 ± 0.7% to 70.7 ± 0.8% (*P* < 0.001). Novobiocin moderately increased AldeRed588 retention in CT26 cells, increasing ALDH positivity from 1.1 ± 0.4% to 22.4 ± 12.7% (*P* < 0.001), but there was no effect on HT29 cells (Figs 3 and 4).

### Influence of Aldefluor assay buffer on ALDH positivity

Assuming that Aldefluor assay buffer contains ABC transporter inhibitors, its ability to suppress reagent efflux was tested. FACS analysis performed in this buffer substantially increased Aldefluor retention in both CT26 (Fig. 3) and HT29 cells (Fig. 4). This raised the ALDH-positive rate from 6.3 ± 2.4% to 86.1 ± 6.4% for CT26 cells (*P* < 0.001) and from 11.0 ± 5.2% to 94.4 ± 0.4% for HT29 cells (*P* < 0.001). Interestingly, AldeRed588 retention was completely uninfluenced by buffer in CT26 cells (Fig. 3) but was substantially increased in HT29 cells (Fig. 4). Thus, ALDH-positive HT29 cells markedly increased from 0.7 ± 0.7% to 63.9 ± 18.8% (*P* < 0.001). The effects of each ABC transporter inhibitor and Aldefluor assay buffer on ALDH positive rates based on Aldefluor and AldeRed588 retention are compared in Figs 3C and 4C. Addition of any of the 3 ABC transporter inhibitors did not additionally increase ALDH positive cells above that caused by Aldefluor assay buffer alone (Supplementary Fig. S5).

### Influence of ABC transporter inhibitors on tumor-sphere formation by ALDH-positive cells

**Figure 5:**
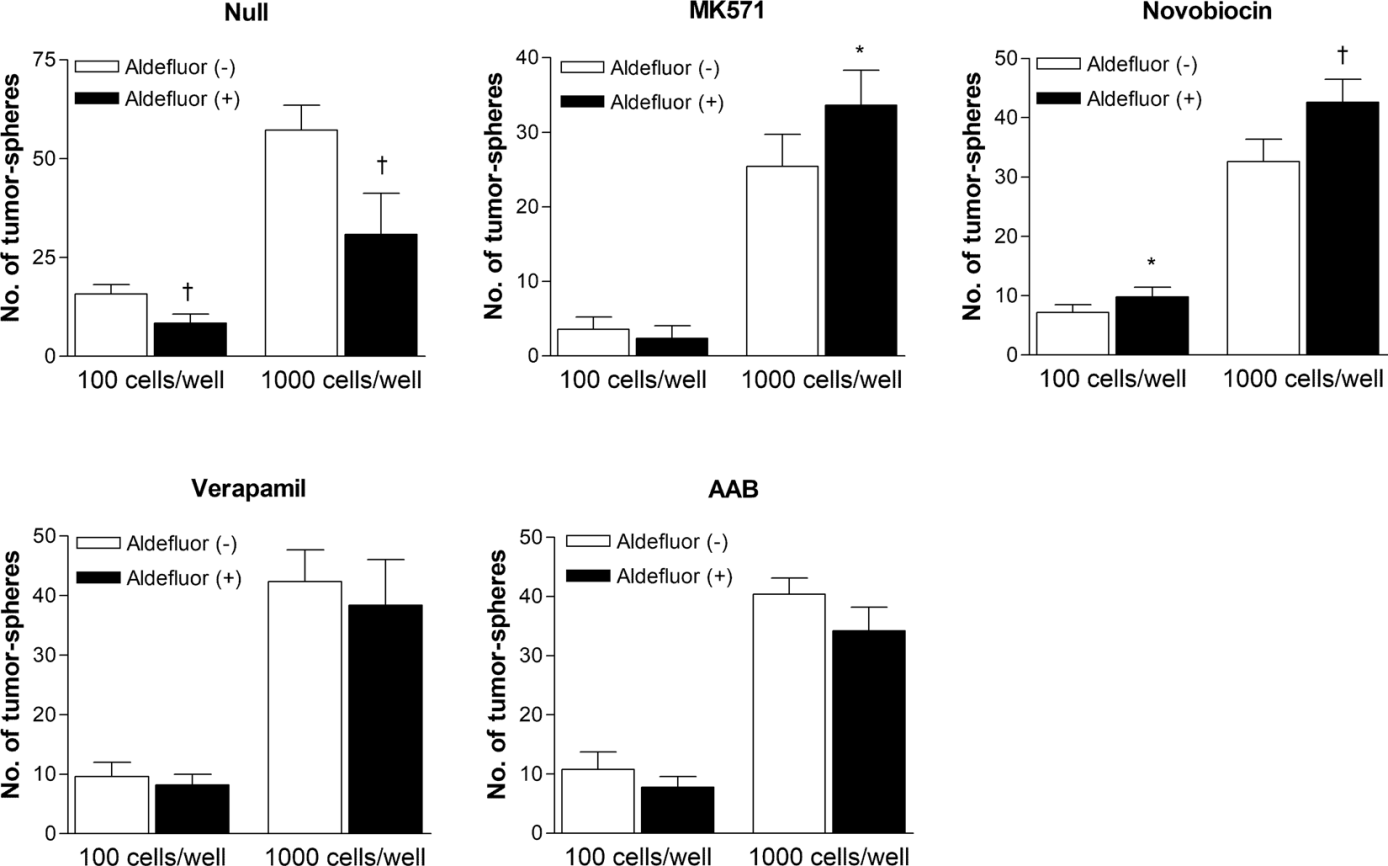
Limiting dilution assays for assessment of functional stemness. Number of tumor-spheres formed 10 days after seeding 100 or 1,000 Aldefluor-negative or -positive CT26 cells selected in buffer containing DMSO vehicle (null), MK571, novobiocin or verapamil, or in AAB. Bars are mean ± SD of % relative to controls obtained from a single experiment with 5 samples per group. ^†^*P* < 0.005, ^*^P < 0.05, compared to wells seeded with identical numbers of Aldefluor-negative cells.

Limiting dilution assays were performed to assess whether Aldefluor-positive cells selected in buffers containing ABC transporter inhibitors had increased functional stemness. The results revealed significantly greater numbers of tumor-spheres with Aldefluor-positive compared to Aldefluor-negative CT26 cells selected in the presence of MK-571 or novobiocin. This was in contrast to cells selected in the absence of any ABC transporter inhibitor (Fig. 5). However, there no significant difference in the number of tumor-spheres formed between Aldefluor-positive and -negative cells selected in buffer containing verapamil or in Aldefluor assay buffer (Fig. 5).

## Discussion

Recent recognition that ALDH plays a central role in tumor progression and stemness has made it important to accurately measure the activity of this enzyme. To perform precise ALDH assays using presently available reagents and to develop newer assay reagents, a clear understanding of how individual ABC transporters influence their efflux from cancer cells is needed.

Efluxx-ID Green assays were used to yield measurements of transporter activity, which are more relevant to substrate efflux capacity than simple assessments of protein amount. The results demonstrated that CT26 cells have significant MDR1, MRP1/2, and BCRP activity, whereas HT29 cells have significant MRP1/2 and BCRP activity. These results are consistent with previous reports of MDR1, MRP2, and BCRP protein in CT26 cells^20^. Similarly, HT29 cells have shown significant amounts of MRP1/3^21^ and BCRP protein^22^, whereas MDR1 expression was low^23^. Western blots also confirmed that both cell types expressed more than one ABC transporter. Although it would have been useful to work with cells that express a single ABC transporter, it was difficult to select such cancer cells with certainty. This is exemplified by a previous mRNA expression profile study demonstrating that virtually all cancer cell lines of the NCI-60 panel express multiple ABC transporters^24^.

Expression of multiple transporters by the cells in this study raises the issue of inhibitor specificity. In concentrations used in this study, the 3 inhibitors are indicated to be specific for MDR1, MRP1/2, and BCRP, respectively^25^. Inhibitor specificity is also supported by a recent study that showed efflux suppression in ovarian cancer cells by MK-571 and novobiocin correlated with greater expression of MRP1 and BCRP, respectively^26^. Another study showed that novobiocin inhibited BCRP but not MDR1 in breast cancer cells^27^. Verapamil was reported to inhibit efflux through MDR1 but not MRP, whereas its ability to inhibit BCRP was not described^25^. However, our efflux assays show that 20 μM of verapamil does not inhibit efflux in MRP- and BCRP-expressing HT29 cells even though it substantially inhibits efflux in MDR1-expressing CT26 cells. Although inhibitor specificity against ABC transporters should be considered, the use of cells that express multiple ABC transporters is not likely to have significantly influenced the major findings of our study.

Although ALDH1A1 has generally been believed to be responsible for the ALDH activity of CSCs, more recent experiments indicate that other ALDH isoforms significantly contribute to Aldefluor positivity in a cancer type-specific manner^10^. In particular, ALDH1A3 and ALDH3A1 are gaining interest as additional markers that may significantly contribute to CSC ALDH activity^28,29^. All of these ALDH isoforms influence CSC biology and also have potential prognostic applications in cancer^10^.

The first paper reporting the utility of BAAA to identify ALDH-positive stem cells used a buffer containing verapamil as an MDR1 inhibitor^30^. The more recently introduced AldeRed ALDH assay kit (Merck Millipore, Germany) uses AldeRed588 as a red-shifted fluorescent ALDH substrate that is synthesized by BODIPY 576/589 succinimidyl ester conjugated to aminoacetaldehyde diethyl acetal^31^. This kit also includes verapamil as a component of assay buffer. We expected that verapamil would suppress efflux of Aldefluor and AldeRed588 products from cells with MDR1 activity. However, the low retention of these reagents in both CT26 and HT29 cells shown at baseline did not increase due to verapamil. This finding is not inconsistent with the patent results for BAAA, in which verapamil caused only a minor incremental increase in Aldefluor positive cells from 0.3% to 1%^32^. Our results indicate that both Aldefluor and AldeRed588 products are poor substrates for MDR1-mediated efflux.

In contrast, treatment with MK-571 caused a marked increase in Aldefluor-based ALDH-positivity in both CT26 and HT29 cells. MK-571 also caused a marked increase of AldeRed588-based ALDH-positive HT29 cells. This shows that MRP is a major transporter through which Aldefluor and AldeRed588 products efflux out of cancer cells. The Aldefluor kit manual states that probenecid, which is considered an MRP inhibitor^33^, can be added for assays, although the circumstances when this is necessary are unspecified. Our results indicated that probenecid or other MRP inhibitors should be added for assays of cells with MRP expression. The Aldefluor kit manual also states that 2-deoxy-D-glucose can be added for assays. Although this agent is generally thought to reduce drug resistance-related efflux, the precise mechanism of action remains unclear^34^. Meanwhile, the AldeRed ALDH Assay kit manual does not mention the use of transporter inhibitors other than verapamil, which is insufficient for MRP-expressing cells.

Increased background fluorescence of cells under different assay buffers (that do not contain fluorescent substrates) might influence FACS results. When this possibility was tested, however, adding DEAB did not influence the fluorescent signals of cells on FACS assays. MK571, novobiocin, and Aldefluor assay buffer mildly increased fluorescence background, but the background fluorescent signal profiles were virtually identical whether or not DEAB was present (Supplementary Fig. S6). Since ALDH positive cells are detected by increased fluorescence in the absence of DEAB, the effects observed with ABC transporter inhibitors and Aldefluor assay buffer were not caused by increased background or auto-fluorescence.

Interestingly, MK-571 markedly increased AldeRed588 retention in HT29 cells, but the effect was only mild in CT26 cells. Although the cause for this is unclear, MRP subtype differences may have contributed. CT26 cells are reported to express MRP2^20^, whereas HT29 cells are known to express MRP1 and MRP3^21^. Differences in MRP subtype are not likely to influence MK-571 action, since this agent inhibits MRP1, 2, 3, and 4. Rather, it is more likely that AldeRed588 product efflux is MRP subtype-dependent and is more efficient for MRP1 and/or MRP3 but less efficient for MRP2. In comparison, Aldefluor product efflux may be efficient for MRP1, 2, and 3.

When the effect of novobiocin was tested, there was a moderate increase in ALDH-positive CT26 cells based on Aldefluor or AldeRed588. In contrast, little effect was observed for HT29 cells. At first glance, the effects of ABC inhibitors on ALDH assays appear less that consistent with Western blot results. However, it should be recognized that the band intensities in our Western blots fail to reflect ABC transport activity and also have limited usefulness for comparing ABC transporter levels. Indeed, the amount of any two ABC transporters cannot be compared because they are blotted using different antibodies. The amount of the same ABC transporter cannot be compared in murine and human cells because the antibody likely binds human and murine protein with different affinities. This was illustrated when Western blots of protein from CT26 cells with an antibody specific for both murine and human BCRP resulted in faint protein bands, but repeating the Western blot with an antibody specific for murine BCRP detected some bands that matched the expected size of the murine protein (Supplementary Fig. S7).

The apparent inconsistencies mentioned above might have been due to inhibitor cross-reactivity with other ABC transporters. There are previous reports that verapamil exerts non-specific inhibition of BCRP^35,36^. In our results, verapamil did not influence Aldefluor efflux in BCRP(+)/MDR1(−) HT29 cells, indicating the absence of significant BCRP inhibition. We did not find any reports for nonspecific inhibition of MDR1 or MRP by novobiocin. Therefore, the inhibitory effect of novobiocin on Aldefluor efflux in CT26 cells likely represents blocking of BCRP activity. This was further supported by our finding that an alternative BCRP inhibitor, Ko143, substantially increased ALDH positivity of CT26 cells and to a lesser extent HT29 cells. The difference in ALDH positivity compared with novobiocin suggests that sensitivity to specific BCRP inhibitors might differ according to cell type.

Finally, since Aldefluor assay buffer likely contains one or more ABC inhibitors, we investigated its effect on reagent retention. Compared to PBS with 2% FBS, the use of Aldefluor assay buffer substantially increased Aldefluor retention in both CT26 and HT29 cells and AldeRed588 retention in HT29 cells. The potency of these effects was similar to those achieved by MK-571, indicating that Aldefluor assay buffer likely contains an MRP inhibitor. In contrast, since the effects were hugely divergent from those obtained with novobiocin, Aldefluor assay buffer is not likely to contain a BCRP inhibitor.

Consistent with the FACS results, microscopic inspection confirmed positive fluorescence in the majority of both HT29 and CT26 cells in Aldefluor assay buffer, although the fluorescence intensity of individual cells was significantly stronger in HT29 cells (data not shown). This supports the notion that most HT29 and CT26 cells are indeed ALDH positive.

Aldefluor-positive cells selected in the presence of MK-571 or novobiocin formed greater numbers of tumor-spheres compared to Aldefluor-negative cells. This indicates that Aldefluor assays may need to be performed in buffer containing one or both of these ABC transporter inhibitors to better select cancer cells with increased functional stemness. There was no difference in the number of tumor-spheres formed between Aldefluor-positive and -negative cells selected in buffer containing verapamil or in AAB. This suggests that, depending on cell type, ALDH expression alone may not be sufficient to ensure greater functional stemness.

ABC transporters are also found in certain differentiated cancer cells. Therefore, inhibition of ABC transporters could also increase Aldefluor retention in cancer cells that express both ALDH and ABC transporters. However, since ALDH activity is higher in CSCs, the extent of Aldefluor retention by ABC transporter inhibition should be greater in these cells than in differentiated cancer cells. Further investigations are thus warranted to establish an optimal threshold for Aldefluor assays performed with appropriate ABC transporter inhibitors and to explore whether this might allow differentiation of CSCs from differentiated cancer cells with greater accuracy. Whether cells isolated using different assay conditions differed in functional stemness is also an interesting question. However, this was beyond the scope of the present study, and further investigations are required to elucidate this issue.

In conclusion, our study clearly demonstrated that MRP and BCRP activities efficiently mediated efflux of Aldefluor and AldeRed588 products. Fluorescent signals reduced by this effect resulted in substantial underestimation of the amount of ALDH-positive cancer cells. This finding underscores the importance of complete blockade of these transporters for accurate assessment of cancer cell ALDH activity and to develop new ALDH assay reagents.

## Materials and Methods

### Cell culture

CT26 mouse colon cancer cells and HT29 human colon cancer cells from the American Type Cell Culture were maintained in RPMI-1640 media (Lonza, Swiss) supplemented with 10% FBS (Serana, Germany), 2 mM L-glutamine, and 100 U/ml penicillin-streptomycin in 5% CO_2_ at 37 °C.

### ABC transporter assay

The activities of major ABC transporters were assessed by Efluxx-ID Green assays (ENZO Life Sciences, Lo¨rrach, Germany) following the manufacturer’s protocol. Briefly, cells harvested by trypsination were pre-incubated for 5 min at room temperature in PBS containing 2% FBS. The buffer contained one of three major ABC transporter inhibitors. Concentrations were 20 μM for the MDR1 inhibitor verapamil (in DMSO), 50 μM for the MRP1/2 inhibitor MK-571 (in DMSO), and 200 μM for the BCRP inhibitor novobiocin (in DMSO). The same concentrations of inhibitors were used for both CT26 and HT29 cells in all experiments. After Efluxx-ID Green dye was added, cells were incubated for 30 min at 37 °C. Finally, 10,000 cells per sample underwent FACS analysis on a Calibur flow-cytometer using CellQuest software (Becton-Dickinson, NJ). Efluxx-ID Green was excited at 490 nm and fluorescence emission was detected at 514 nm.

### Western blotting for ABC transporters and ALDH

All antibodies against ABC transporters and ALDH isoforms that were used for immunoblotting have reactivity against antigens of both human and murine proteins. However, BCRP immunoblot for CT26 cells was performed with a murine-specific antibody because the antibody with reactivity against antigens of both human and murine proteins showed only faint BCRP bands in these cells. Cells were washed with PBS and solubilized in Pro-Prep protein extraction solution (iNtRON Biotechnology, Korea) for 15 min at 4 °C, and cell debris was eliminated by centrifugation at 14,000 rpm for 10 min at 4 °C. The supernatant was analyzed for protein content by the Bradford method, and 20–60 μg of protein were separated on an 8% polyacrylamide gel for ABC transporter detection and on a 10% polyacrylamide gel for ALDH detection. Protein was transferred to a hydrobond ECL nitrocellulose membrane (Amersham Biosciences, NJ) and incubated overnight at 4 °C with polyclonal antibodies against ALDH1A1 (Abcam, MA; 1:1000), ALDH1A2 (Abcam; 1:1000), ALDH1A3 (Genetex, CA; 1:1000), ALDH3A1 (Cusabio, China; 1:1000), MDR1 (Abcam; 1:1000), MRP1 (Abcam; 1:250), MRP2 (Abcam; 1:1000), MRP3 (Abcam; 1:250), and BCRP in tris-buffered saline (50 mM Tris, pH 7.5, 150 mM NaCl) containing 0.05% polysorbate-20 and 5% skim milk. The antibody for BCRP immunoblot was from Abcam for human cells (1:1000) and from NSJ Bioreagent (CA) for murine cells (1:500). After washing 3 times for 10 min with tris-buffered saline with Tween 20, the membrane was incubated with secondary antibodies for 1 h at room temperature. Immune reactive protein was finally detected with an enhanced chemiluminescence kit (Thermo Fisher Scientific, MA).

### ALDH assays with major ABC transporter inhibitors

Cellular ALDH activity was assayed using Aldefluor (Stemcell Tech. BC) or AldeRed588 reagents. Since components of the Aldefluor assay buffer (Stemcell Tech. BC) are proprietary, assays were performed in PBS containing 2% FBS unless otherwise specified. Separate sets of cells underwent assays in RPMI media or HBSS containing 2% FBS. Cells were incubated with 1 μM Aldefluor or AldeRed588 at 37 °C and 5% CO_2_ for 30 min in a humidified atmosphere. For negative controls, the same concentrations of ABC transporters were used while ALDH activity was blocked with 15 μM of the selective ALDH inhibitor DEAB. The same amount of vehicle as in treatment groups was added to all null groups. After incubation, cells were washed and underwent FACS analysis as above. Aldefluor oxidation products were detected by a combination of 488 nm laser as the excitation channel and a 530-nm wavelength fluorescence emission detector channel. AldeRed588 oxidation products were detected by a combination of green 532-nm laser as excitation and 585/42-nm as the wavelength detector channel. ALDH positive cells in each inhibitor-treatment group were identified by fluorescence exceeding the region for control cells that were under identical conditions but with DEAB.

Although the Aldefluor kit may have originally been designed for human cells, it is also widely used to detect ALDH activity as a stem cell marker in murine cells^37^, including CT26 cells^16,38^.

### Limiting dilution assay

CT26 cells were sorted using Aldefluor FACS assays. The upper and lower 10% of cells (or maximum number within regions) were selected as Aldefluor-positive and -negative cells, respectively. After sorting, 10, 100, or 1,000 cells were seeded onto a 96-well ultra-low attachment culture plate (Corning, NY) in CSC selection media (DMEM/F12 base containing 0.4% bovine serum albumin, 5 ml/l B27 (Gibco, MA), 5 μg/ml bovine insulin, 4 μg/ml heparin, 20 ng/ml fibroblast growth factor 2, and 20 ng/ml epidermal growth factor). The number of tumor-spheres, defined as cell aggregates over 50 μm in diameter, was counted after 10 days of incubation.

### Statistical analysis

Data are presented as mean ± SE of samples. Significant differences between groups were analyzed by Student t-tests for 2 groups and ANOVA with Tukey post hoc tests for 3 or more groups. *P* values < 0.05 were considered statistically significant.

## Supplementary information

Supplementary info

## Data Availability

All data generated or analyzed during this study are included in this published article.
