# Supplementary material for: ATP-binding Cassette Transporters Substantially Reduce Estimates of ALDH-positive Cancer Cells based on Aldefluor and AldeRed588 Assays
Source: Sci Rep. 2019 Apr 23;9:6462. doi: 10.1038/s41598-019-42954-9 (PMC6478741; doi:10.1038/s41598-019-42954-9)
Supplement: Supplementary file 1 — Supplementary info [file 41598_2019_42954_MOESM1_ESM.docx]

**Supplementary information**

**ATP-binding Cassette Transporters Substantially Reduce Estimates of ALDH-Positive Cancer Cells Based on Aldefluor and AldeRed588 Assays**

**(Short Title: ABC Transporter Influence on ALDH Assays)**

Jin Won Park*^1,2^*, Kyung-Ho Jung*^1,2^*, Youngjoo Byun*^3^*, Jin Hee Lee*^1,2^*,

Seung Hwan Moon*^1^*, Young Seok Cho*^1^*, Kyung-Han Lee*^1,2^*^,*^

^1^Department of Nuclear Medicine, Samsung Medical Center, Seoul, Korea,

^2^Department of Health Sciences and Technology, SAIHST, Sungkyunkwan University School of Medicine, and ^3^College of Pharmacy, Korea University, Korea

*Corresponding Author (For reprints): Kyung-Han Lee, MD, PhD.

Department of Nuclear Medicine, Samsung Medical Center,

50 Ilwon-dong, Gangnam-gu, Seoul, Korea.

Tel : 82-2-3410-2630; Fax : 82-2-3410-2639; [khnm.lee@samsung.com](mailto:khnm.lee@samsung.com)

**
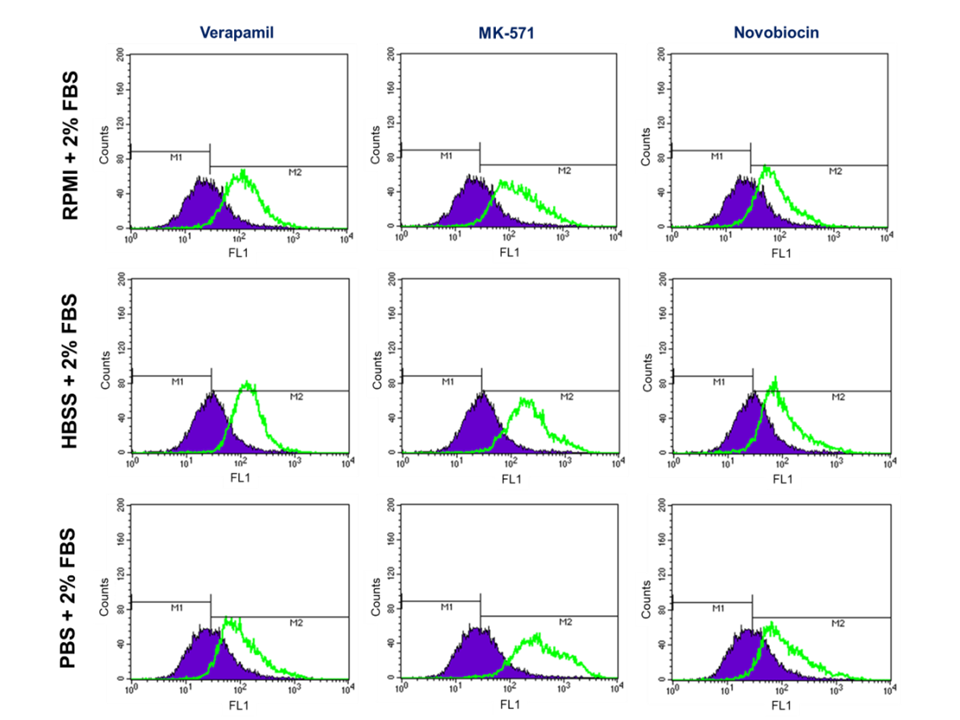
**

**Supplementary Figure S1:** **Effect of buffer on Efluxx-ID Green transporter assays.** Histograms of CT26 cells at baseline (purple) and after treatment (green) with verapamil, MK-571 or novobiocin as specific inhibitors of MDR1, MRP1/2 and BCRP activity, respectively. Assays were repeated in PBS, HBSS or RPMI media containing 2% FBS (representative of 3 samples per group).

**
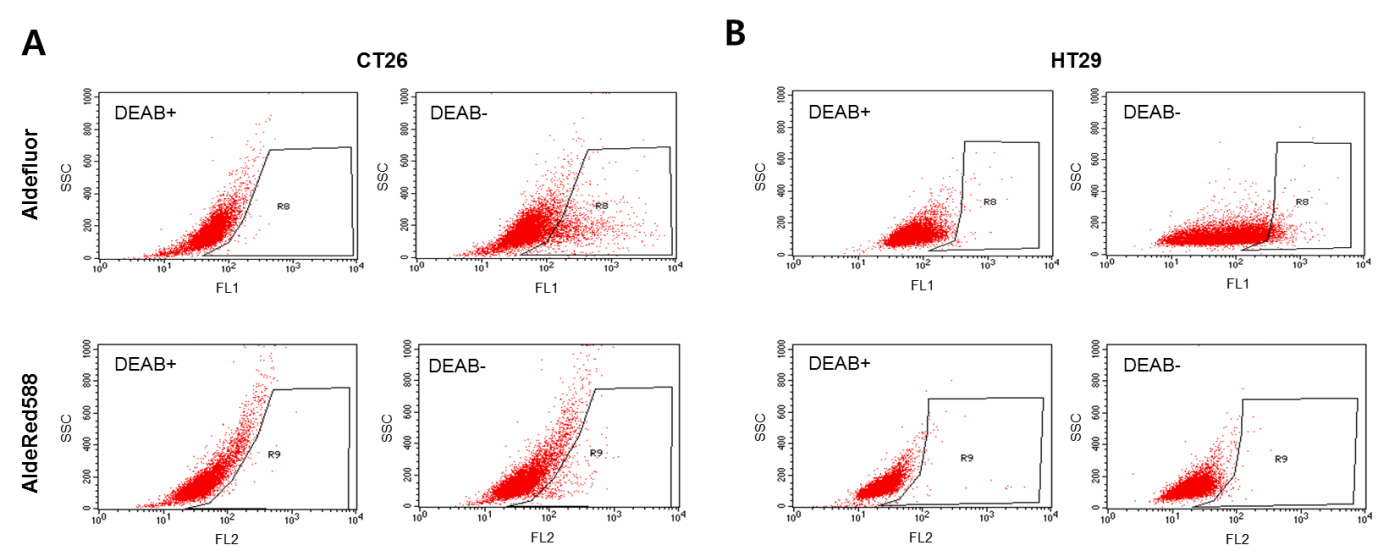
**

**Supplementary Figure S2:** **Identification of ALDH positive cells by FACS using Aldelfluor and AldeRed588.** Representative ALDH assays of CT26 cells **(A)** and HT29 cells **(B)** based on Aldelfluor (top) and AldeRed588 product retention (bottom) in the presence or absence of 15 μM DEAB. The distribution of cells in the presence of DEAB were used to define ALDH-positive cells (representative of 4-6 samples per group).


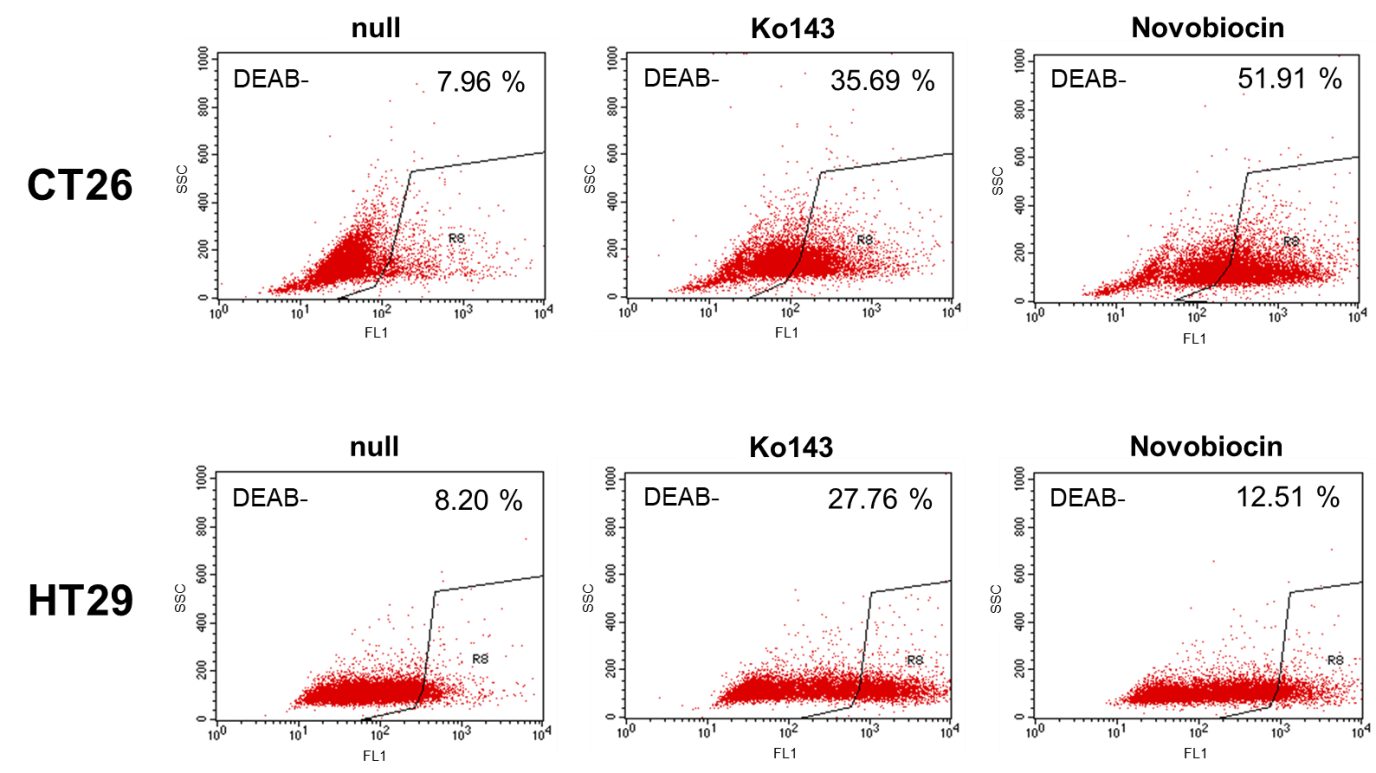


**Supplementary Figure S3: Comparison between BCRP inhibitors in CT26 and HT29 cells.** ALDH positivity of CT26 and HT29 cells compared to the Ko143 (5 μM) and novobiocin (200 μM) in Aldefluor assay (representative of 2 samples per group)..

**
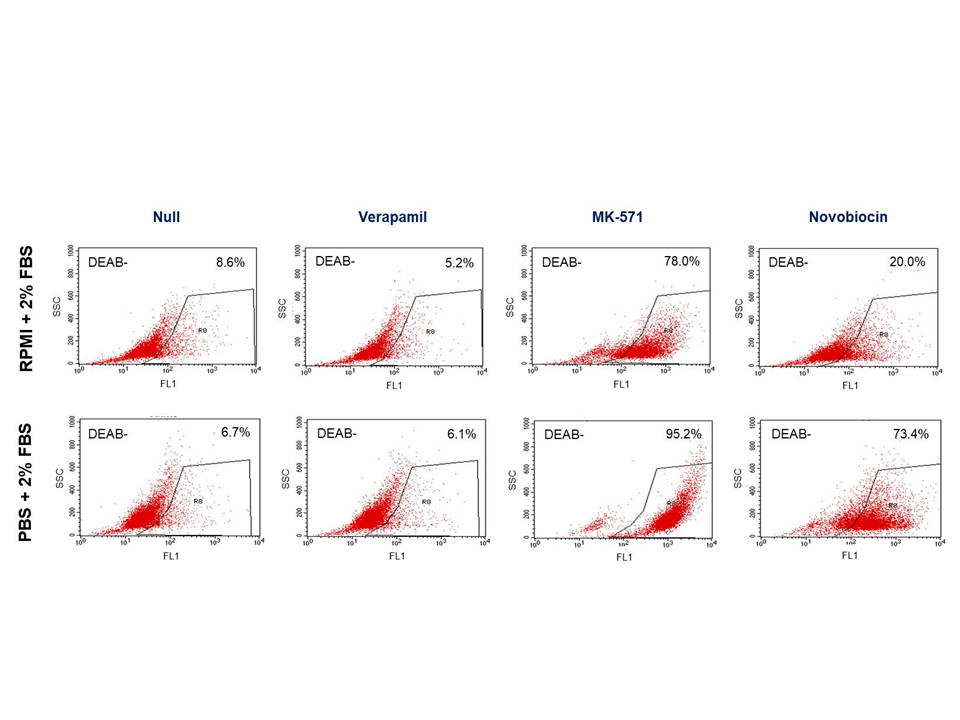
**

**Supplementary Figure S4: Effect of buffer on Aldelfluor efflux in the presence of ABC transporter inhibitors.** Comparison of Aldelfluor assay results of CT26 cells at baseline and in the presence of verapamil, MK-571 or novobiocin, when performed in RPMI media (top) or PBS (bottom) containing 2% FBS (representative of 2 samples per group).


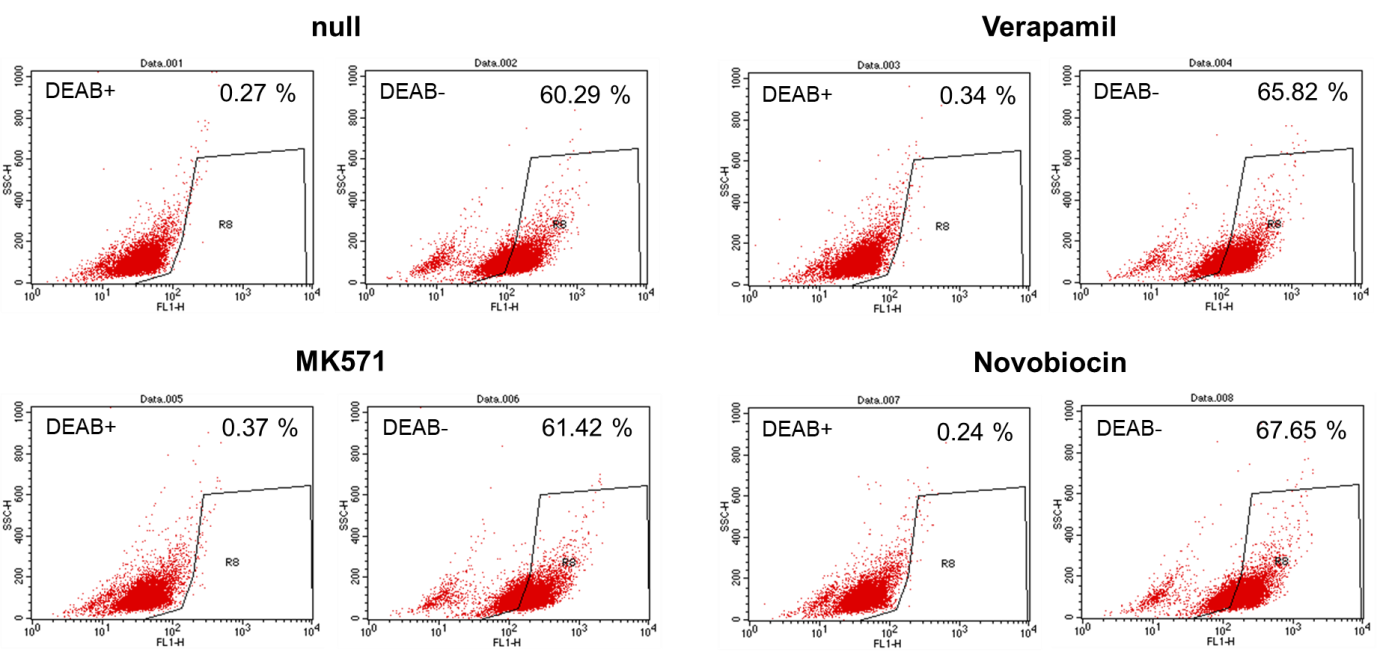


**Supplementary Figure S5: Addition of ABC transporter inhibitors to AAB did not further increase the rate of ALDH positive cells in FACS assays.** Representative ALDH positive cell of the verapamil (20 μM), MK571 (50 μM) and novobiocin (200 μM) compared to Aldefluor assay buffer alone (representative of 2 samples per group). Null condition indicates Aldefluor assay buffer with DMSO vehicle alone.

**
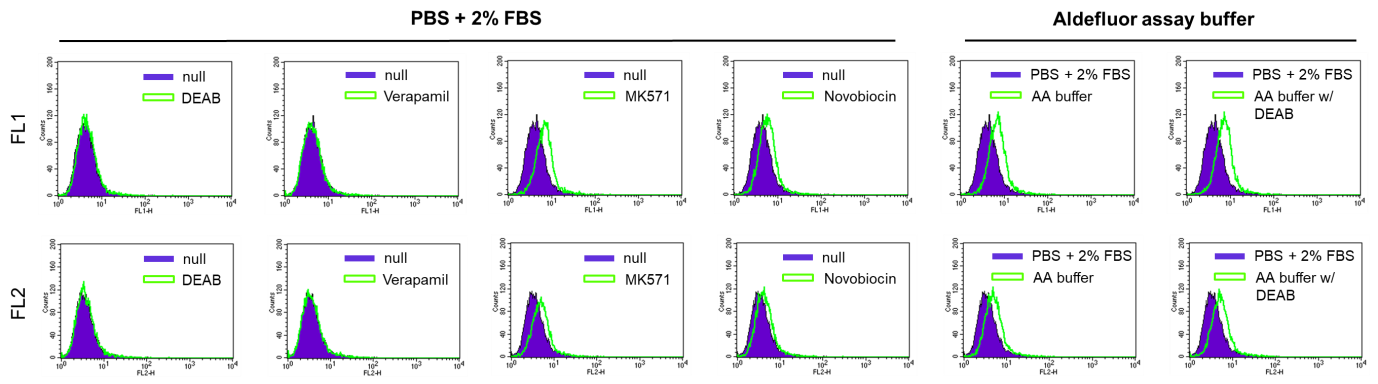
**

**Supplementary Figure S6: DEAB and ABC inhibitors do not influence the auto-fluorescent background signal in FACS assays.** Representative auto-background fluorescence signals of DEAB (15 μM), verapamil (20 μM), MK571 (50 μM) and novobiocin (200 μM) compared to vehicle (DMSO) alone (representative of 2 samples per group). Comparisons are also shown between AAB and PBS with 2% FBS. Neither Aldefluor nor AldeRed588 substrate was added. FL1 and FL2 are wavelengths for Aldefluor and AldeRed588.

**
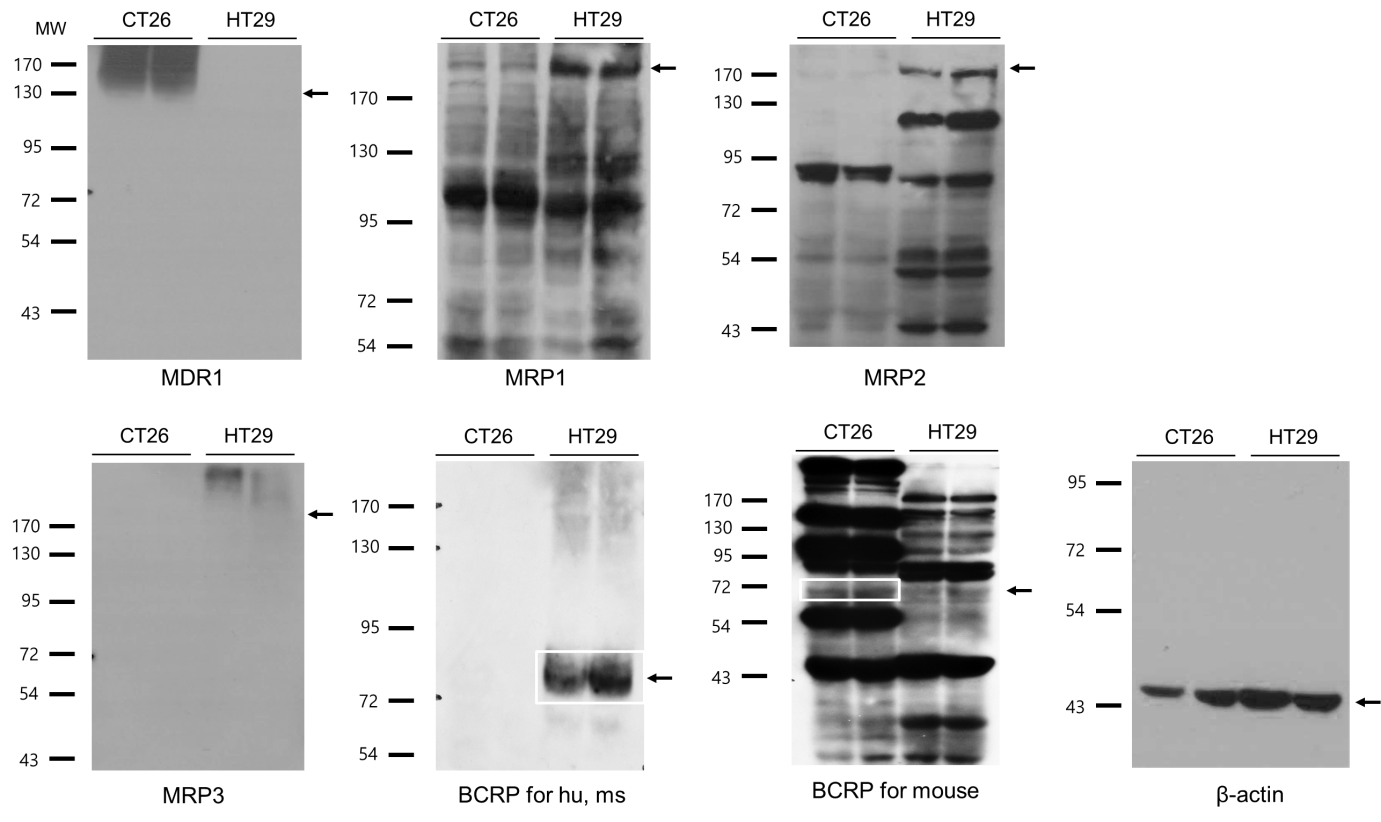
**

**Supplementary Figure S7: Full length blots from Figure 1B.** Western blots of protein from cell lysate for detection of MDR1, MRP1, MRP2, MRP3, BCRP and β-actin (after stripping of BCRP antibodies). MW, molecular weight in kDa.

**
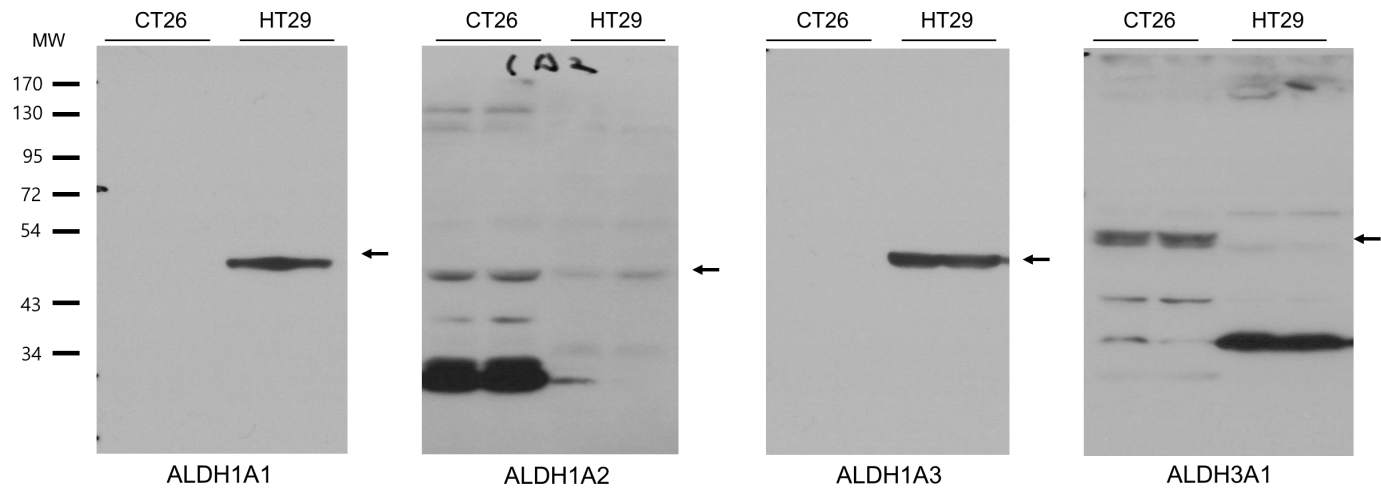
**

**Supplementary Figure S8: Full length blots from Figure 2.** Western blots of protein from cell lysate for detection of ALDH1A1, ALDH1A2, ALDH1A3 and ALDH3A1. MW, molecular weight in kDa.
